# Understanding health data social licence: An international comparison of community attitudes towards health data use across Canada and Australia

**DOI:** 10.23889/ijpds.v9i5.1659

**Published:** 2024-09-18

**Authors:** Julia Burt, Kate Miller, Felicity Flack

**Affiliations:** 1 Health Data Research Network Canada; 2 Population Health Research Network (Australia)

## Objective

Research has found general but conditional support for health data being used for public benefit. The term “social licence” describes which uses of health data the public supports and under what conditions. Here, we aim to compare two approaches to understanding community attitudes towards health data use, and how social licence may differ, between Canadian and Australian populations.

## Approach

Factors that affect community support for health data use include the specific population, the type(s) of data, and the engagement approach used. In Canada, facilitated dialogues were held to explore whether (i) there were uses of health data that diverse members of the public all supported and (ii) there was consensus on essential requirements for health data social licence.

In Australia, national surveys and citizens’ juries were conducted to better understand (i) attitudes towards private sector data use and (ii) the ethical, legal and social implications of using general practice data in research.

## Results

Despite the different approaches taken, many conditions for social licence were similar across Canadian and Australian participants. Both groups agreed on conditions for health data social licence related to equity, governance, privacy and transparency. However, there was a stark contrast between levels of support for private sector data use, personal control and consent.

## Conclusion

This comparative exercise contributes valuable insights into the ongoing dialogue surrounding community attitudes towards health data use. Continued research monitoring health data social licence across populations is imperative for public trust while gaining full benefits from health data use in research.

